# Public Health Impact and Cost-Effectiveness of Screening for Active Tuberculosis Disease or Infection Among Children in South Africa

**DOI:** 10.1093/cid/ciad449

**Published:** 2023-08-05

**Authors:** Joseph Brough, Leonardo Martinez, Mark Hatherill, Heather J Zar, Nathan C Lo, Jason R Andrews

**Affiliations:** National Capital Consortium, Walter Reed National Military Medical Center, Bethesda, Maryland, USA; Department of Epidemiology, School of Public Health, Boston University, Boston, Massachusetts, USA; South African Tuberculosis Vaccine Initiative, Institute of Infectious Disease and Molecular Medicine, and Division of Immunology, Department of Pathology, University of Cape Town, Cape Town, South Africa; Department of Pediatrics and Child Health, Red Cross War Memorial Children's Hospital and SA-MRC Unit on Child & Adolescent Health, University of Cape Town, Cape Town, South Africa; Division of Infectious Diseases and Geographic Medicine, Stanford University School of Medicine, Stanford, California, USA; Division of Infectious Diseases and Geographic Medicine, Stanford University School of Medicine, Stanford, California, USA

**Keywords:** tuberculosis, pediatrics, contact investigations, modeling, preventive treatment

## Abstract

**Background:**

Although tuberculosis disease is a leading cause of global childhood mortality, there remain major gaps in diagnosis, treatment, and prevention in children because tuberculosis control programs rely predominantly on presentation of symptomatic children or contact tracing. We assessed the public health impact and cost-effectiveness of age-based routine screening and contact tracing in children in South Africa.

**Methods:**

We used a deterministic mathematical model to evaluate age-based routine screening in 1-year increments from ages 0 to 5 years, with and without contact tracing and preventive treatment. Screening incorporated symptom history and tuberculin skin testing, with chest x-ray and GeneXpert Ultra for confirmatory testing. We projected tuberculosis cases, deaths, disability-adjusted life years (DALYs), and costs (in 2021 U.S. dollars) and evaluated the incremental cost-effectiveness ratios comparing each intervention.

**Results:**

Routine screening at age 2 years with contact tracing and preventive treatment averted 11 900 tuberculosis cases (95% confidence interval [CI]: 6160–15 730), 1360 deaths (95% CI: 260–3800), and 40 000 DALYs (95% CI: 13 000–100 000) in the South Africa pediatric population over 1 year compared with the status quo. This combined strategy was cost-effective (incremental cost-effectiveness ratio $9050 per DALY; 95% CI: 2890–22 920) and remained cost-effective above an annual risk of infection of 1.6%. For annual risk of infection between 0.8% and 1.6%, routine screening at age 2 years was the dominant strategy.

**Conclusions:**

Routine screening for tuberculosis among young children combined with contact tracing and preventive treatment would have a large public health impact and be cost-effective in preventing pediatric tuberculosis deaths in high-incidence settings such as South Africa.

## Teaser

Using mathematical modelling, we provide evidence that age-based routine screening in combination with contact screening in high-burden tuberculosis regions may be cost-effective in reducing pediatric tuberculosis morbidity and mortality.

## Graphical Abstract

**Graphical Abstract:**
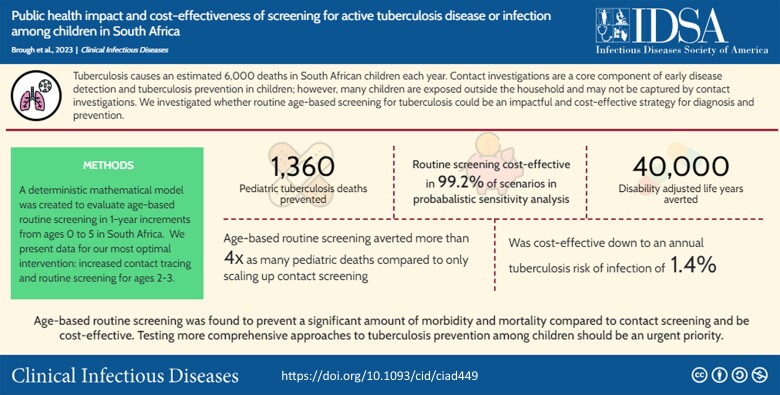

https://tidbitapp.io/tidbits/public-health-impact-and-cost-effectiveness-of-screening-for-active-tuberculosis-disease-or-infection-among-children-in-south-africa

Tuberculosis (TB) is among the leading causes of child mortality globally [1]. In 2020, more than 1 million children younger than age 16 years developed TB disease, and an estimated 225 000 children died of TB and its complications [2]. TB treatment is highly effective among children, with case fatality among treated cases of less than 1% [3]. Most TB deaths in children occur from lack of diagnosis and treatment [4]. In South Africa, the majority of cases in young children younger than age 5 years are estimated to remain untreated [2]. To reduce morbidity and mortality of TB among children, a rigorous evaluation of strategies for case detection and prevention is needed.

An overarching approach to deal with the pediatric TB epidemic is not well established. Contact tracing in households of TB cases is among the central strategies recommended by the World Health Organization for early case detection and preventive treatment among children [5–9]. Previous studies have found contact tracing to be cost-effective in countries with high TB incidence [10]. However, emerging evidence suggests that TB infection among children is often acquired outside the household [6, 7, 11]; therefore, household contact tracing alone may be insufficient as a population-based approach for the pediatric TB epidemic. Population-based screening approaches could reach more children; however, it is unclear whether such broad screening would be cost-effective.

To further understand the effectiveness and cost implications of distinct interventions when applied at a population level in a high-burden setting, we developed a deterministic mathematical model of pediatric TB in South Africa [12]. We investigated the public health impact and cost-effectiveness of routine screening for TB infection and disease among children during the period they are most susceptible to progress from TB infection to disease: from ages 0 to 5 years [13], as well as household contact tracing. We further examined the value of preventive treatment to these population-level interventions.

## METHODS

### Overview

We developed a deterministic, mathematical model of pediatric TB natural history, treatment for disease, and preventive treatment, parameterized by natural history and diagnostic data from the published literature. We used epidemiological and cost data from South Africa. We then used this model to evaluate the public health impact and cost-effectiveness of several interventions, including: (1) increased contact screening of household contacts ages 0 to 15 years and (2) age-based routine screening in 1-year increments from ages 0 to 5 years. We compared these interventions with current estimates for TB case detection in South Africa.

### Model Structure and Assumptions

The model consists of 4 TB natural history states: susceptible, early TB infection, late TB infection, and TB disease (detailed further in the Supplementary appendix, Supplementary Figures 1 and 2). All individuals start in the susceptible state at birth and, if infected, enter an early TB infection state for 1 year. Consistent with prior pediatric and adult TB models, early TB infection has a significantly greater rate of progression to disease than a late TB state [14]. We modeled a constant force of infection of 4% derived from local estimates in South Africa [1, 15–17]. We did not model transmission because interventions targeted to children are unlikely to have important transmission impacts over short horizons.

Each natural history and treatment compartment was further stratified by age (ages 0, 1, 2, 3, 4, 5–10, and 10–15 years), human immunodeficiency virus (HIV) status, and Bacille Calmette-Guerin vaccine (BCG) immunization status. Epidemiologic parameters varied by age, BCG, and HIV status (Table 1 and Supplementary Table 1). We modeled treatment and preventive treatment states that could be entered from each natural history state.

**Table 1.** Model Inputs for Natural History, Treatment and Diagnostic Parameters, and Costs for Tuberculosis Screening in Children

Parameter	Value	Confidence Interval in Probabilistic Sensitivity Analysis	Ref
Annual risk of TB infection	4%	…	[2, 15–17]
Proportion of early TB infections that progress to disease	6%–26%^a^	…	[13]
Risk of late TB infection progressing to disease per year	0.07%–1.8%^a^	…	[18]
Risk of TB reinfection per year	21% of annual risk of TB infection	…	[18]
Risk of death from treated TB	0.3%–2%^a^	0.16%–18%^a^	[3]
Risk of death from untreated TB	Age 0–4 y: 38% Age 5+: 10%	Age 0–4 y: 30%–50% 5+: 5%–20%	[19]
Background TB detection (proportion of TB cases detected without any screening interventions)	Age 0–4 y: 75% Age 5–15 y: 70%	Age 0–4 y: 30%–93% Age 5–15 y: 15%–94%	[2, 20, 21]
Isoniazid preventive treatment success and completion rate	59%	40%–80%	[22]
Tuberculin skin test sensitivity	88.2%	65%–96%	[23]
Tuberculin skin test specificity	86.3%	67%–95%	[23]
Symptom screen sensitivity	52%–82%^a^	44%–87%^a^	[24]
Symptom screen specificity	61%–93%^a^	46%–96%^a^	[24]
GeneXpert Ultra sensitivity	72.8%	55%–85%	[25]
GeneXpert Ultra specificity	97.5%	95%–99%	[25]
Chest x-ray sensitivity	50%	16%–84%	[26]
Chest x-ray specificity	85%	70%–95%	[26]
Cost clinic visit (USD)	29.4	25–33.8	[10]
Cost of chest x-ray (USD)	51.5	43.7–59.2	[10]
Cost of tuberculin skin test (USD)	80.0	66.3–89.7	[10]
Cost tuberculosis preventive treatment (USD)	147.8	126–170	[10]
Cost of GeneXpert (USD)	30.3	29–32	[27]
Cost of TB treatment (USD)	Age 0–2 y: 2068 Age 2–15 y: 2635	Age 0–2 y: 1758–2 379a Age 2–15 y: 2240–3 030a	[10]
Proportion of TB infections from household transmission	13.1%	4%–50%	[11]
Proportion of population with BCG vaccination within first year of life	86%	…	[28]
HIV prevalence at birth (% of population)	0.116%	…	[12]
Background death	As per WHO life tables and annual deaths reported in South Africa	…	[29]

Costs are in July 2021 USD.

Abbreviations: BCG, Bacille Calmette-Guerin vaccine; TB, Tuberculosis; USD, United States Dollar; WHO, World Health Organization.

^a^Indicates values differ based on age and/or HIV status and are further described in Supplementary Table 1.

### Base Case

We included a base case (or “status quo”) model that represented the current estimate of both passive tuberculosis case finding and contact screening. Passive case finding (“background detection”) was set at 75% for ages 0 to 4 years and 70% for ages 5 to 15 years [2, 20, 21]. Contact screening with preventive treatment was set at 38% of all pediatric TB contacts [30, 31]. All interventions were compared with this base case.

### Interventions

We evaluated 3 active case finding strategies implemented at the population level: (1) increased contact investigations for all individuals ages 0 to 15 years; (2) age-based routine screening; and (3) increased contact investigations combined with age-based routine screening.

Contact investigations were prompted by diagnosis of TB in the household. Household size and TB contact prevalence were formulated from Demographic Health Survey data [32]. Based on these data and studies regarding adult prevalence of TB in South Africa, at any given time, 2% of all children ages 0 to 15 years were exposed to tuberculosis in the household [11, 15]. Infection rates for household contacts were fitted so that 13% of the total pediatric TB burden was spread by household contacts [11].

In our intervention, 75% of child TB contacts would be prompted for screening, based on real-world vaccine uptake [28]. The screening algorithm is further illustrated in Supplementary Figure 3 in the Supplementary appendix. Children with a household contact were screened for symptoms and sputum Xpert Ultra. If symptom positive and Xpert Ultra negative, a chest x-ray was used. Those either Xpert Ultra positive or with evidence of TB disease on chest x-ray were given therapy for TB disease. All other contacts were given a TB preventive treatment (TPT) regimen of 6 months of daily isoniazid [14, 33]. All interventions used TPT.

Age-based routine screening was modeled in distinct age groups, in 1-year increments from age 0 to age 5 years. We assumed that screening would be performed at time of vaccination or other routine medical interactions. The screening coverage for this intervention was also modeled at 75% [2, 4].

For children without a household contact, screening consisted of symptom screening and tuberculin skin testing with chest x-ray and induced sputum Xpert Ultra if either symptom or tuberculin skin test screening was positive (Supplementary Figure 3). TPT was given to children who screened positive in tuberculin skin testing but negative in Xpert Ultra and chest x-ray. For all interventions, TB disease pharmacotherapy was given to children who screened positive on either Xpert Ultra or chest x-ray. Treatment modalities incorporate real-world effectiveness and completion rates.

### Costs, Disabilities, and Incremental Cost-effectiveness Analysis

We projected costs from a societal perspective, drawing on published literature on diagnostic and treatment costs from South Africa [27]. Costs in our model included clinic visits, test consumables, administration fees, staffing, medications, and management of adverse effects. Costs to patients included transportation costs and time associated with the visit [10]. All costs were measured with U.S. dollars and inflation-adjusted to July 2021.

We projected disability-adjusted life years under each strategy, using utility estimates in which 0 is death and 1 is perfect health. We used the disability weights during undiagnosed TB (0.76) and during anti-TB treatment (0.87) [34]. We assumed a utility of 0 on death and projected life years lost up to the South Africa life expectancy of 64 years. All disabilities and costs were discounted at 3% per year. We calculated incremental cost-effectiveness ratios (ICERs), defined as the difference in costs divided the difference in disability-adjusted life years when comparing 2 strategies, reported in U.S. dollars per disability adjusted life years averted. For broad interpretation, we considered ICERs lower than South Africa's 2020 annual gross domestic product per capita ($6414) to be highly cost-effective, and ICERs lower than 3 times the annual gross domestic product per capita ($19 242) to be cost-effective [35, 36], although these interpretations are not meant to be strict and instead should be guided by a country's priorities to determine the willingness-to-pay threshold.

### Sensitivity and Scenario Analyses

We performed probabilistic sensitivity analysis on uncertain parameters in our model. We performed Latin hypercube sampling to draw 10 000 parameter sets from uncertainty distributions (Table 1), projecting disability-adjusted life years, outcomes, and ICERs for each simulation. We performed 1-way sensitivity analysis on all parameters in the probabilistic sensitivity analysis and a 2-way sensitivity analysis between the proportion of population screened and background detection rate. We performed scenario analysis by varying the force of infection from 0.1% to 10% and by including a scenario that did not include TPT.

## RESULTS

In the base case, 40% of the population had tuberculosis infection by the age of 15 years with 2% of the population having active disease. There were 187 pediatric deaths per 100 000 person-years, 39 per 100 000 person-years related to TB (31 900 deaths in the entire South African pediatric population up to age 15 years, 6600 related to TB). Compared with the base case, all strategies decreased deaths and disability-adjusted life years and were associated with increased costs (Table 2).

**Table 2.** Public Health Impact, Cost, and Incremental Cost-effectiveness of Selected Tuberculosis Case Finding and Prevention Strategies Among Children in South Africa Ages 0–15 y^c^

Intervention^a^	Total Cost, Millions USD (95% CIs)	Cost Greater Than Base Case, Millions USD (95% CIs)	Total DALYs, in 1000s (95% CIs)	DALYs Averted, in 1000s (95% CIs)	Total Deaths (95% CIs)	Total Deaths Averted (95% CIs)	ICER, $ Per DALY (95% CIs)
Base case	350 (151–505)	…	879 (755–1168)	…	31 910 (26 700–44 600)	–	–
Contact screening only	409 (212–572)	59 (43–89)	871 (750–1146)	8 (3–29)	31 640 (26 500–41 000)	270 (60–1150)	7 370^b^
Age-based routine screening, age 2 y	488 (304–653)	138 (111–205)	845 (744–1092)	34 (10–82)	30 760 (26 300–41 000)	1150 (210–3800)	4130 (1940–11 880)
Contact + age-based routine screening, age 2 y	541 (358–715)	191 (156–278)	839 (741–1073)	40 (13–100)	30 550 (26 300–41 000)	1360 (260–3800)	9050 (2890–22 920)

Abbreviations: CI, confidence interval; DALY, disability-adjusted life year; ICER, incremental cost-effectiveness ratio; USD, US dollar.

^a^This table represents a subset of interventions trialed. For a full list of interventions, please see Supplementary Table 2.

^b^Strategy is dominated with the inclusion of other interventions and therefore is not included in the final calculation of ICERs for interventions; ICER is compared only with the base case

^c^All strategies in this table include tuberculosis preventive treatment.

Contact investigation for ages 0 to 15 years at 75% coverage combined with preventive treatment averted 270 deaths (95% confidence interval [CI]: 60–1150), 3300 (95% CI: 1550–6730) TB cases, 8000 disability-adjusted life years (95% CI: 3000–29 000) and cost an additional $59 million (95% CI: 43–89) compared with the base case. The most effective age for age-based screening was age 2 years, in which 1150 deaths (95% CI: 210–3800), 8900 tuberculosis cases (95% CI: 3720–11 690), and 34 000 disability-adjusted life years (95% CI: 10 000–82 000) were averted with an additional cost compared with the base case of $138 million (95% CI: 111–205). If age-based routine screening at age 2 years was combined with contact investigations, the number of deaths, disability-adjusted life years, and TB cases averted compared with the base case increased to 1360 (95% CI: 260–3800), 40 000 (95% CI: 13 000–100 000), and 11 900 (95% CI: 6160–15 730), respectively. The total added cost of this combination intervention also increased to $191 million (95% CI, 156–278).

Contact investigations were cost effective compared with the status quo but were dominated by a strategy of age-based routine screening at age 2 years with an ICER of $4130 per disability-adjusted life years averted (95% CI: $1940–11 880). Contact investigations combined with age-based screening was the second intervention on the cost-effectiveness frontier with an ICER of $9050 (95% CI: 2890–22 920) per disability-adjusted life years averted, dominating all other interventions. This intervention would be categorized as cost-effective, falling below 3 times the 2021 gross domestic product per capita of South Africa ($19 242) [35].

The addition of TPT to contact investigations and routine age-based screening increased the public health impact and decreased the cost-effectiveness compared with interventions that did not include TPT (Supplementary Table 3). When TPT was included, the disability-adjusted life years averted were 48% greater (21 000 disability-adjusted life years [DALYs] averted vs 40 000 DALYs averted) and the ICER was 8% greater (8390$/DALY vs 9050$/DALY). Despite an overall decrease in cost-effectiveness with TPT, preventive treatment was still a cost-effective intervention.

The effectiveness of these interventions were positively associated with the background annual force of TB infection. As the force of TB infection increased, the public health impact and cost-effectiveness of interventions also increased. Age-based routine screening plus contact screening remained cost-effective when the force of TB infection was greater than 1.6%, and routine screening alone without increased contact screening remained cost-effective above a force of infection of 0.8%.

**Figure 1.**
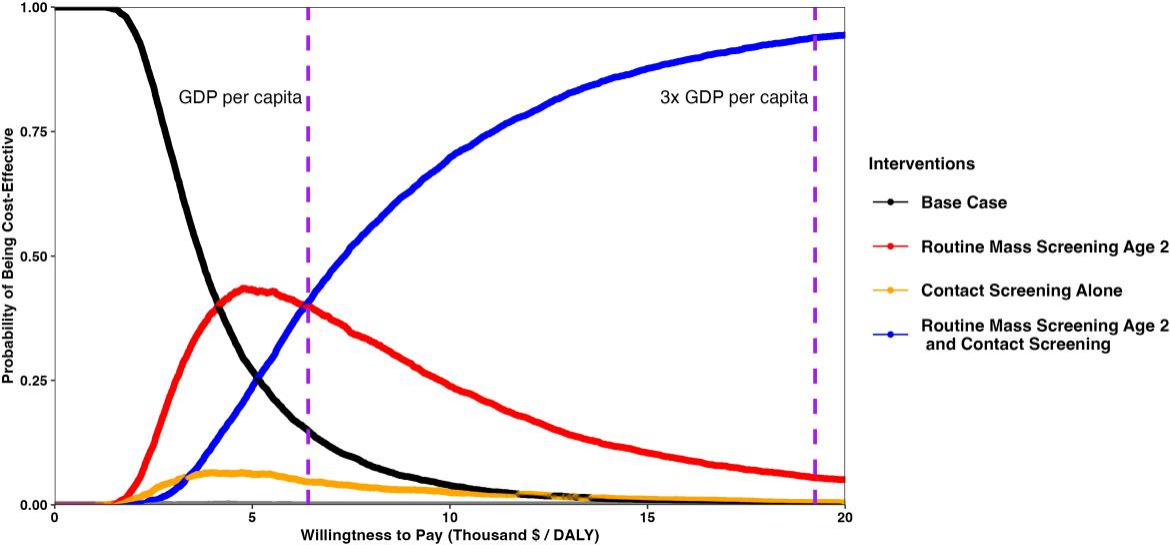
Cost-effectiveness acceptability curve of distinct interventions to prevent and detect pediatric tuberculosis. At 1× GDP per capita of South Africa ($6414), Contact screening combined with routine screening of age 2 years is the dominant intervention on 44% of probabilistic sensitivity analysis runs, with some intervention including routine screening dominating 79% of runs. At 3× GDP per capita, contact screening combined with routine screening is the optimal strategy for 95% of runs, with 99.2% of runs dominated by some form of routine screening. These willingness-to-pay thresholds (vertical purple line) are provided for broad interpretation but are not designed to be strict; the willingness-to-pay threshold should be determined by each country's priorities. Generated from probabilistic sensitivity analysis. All strategies in this figure include tuberculosis preventive treatment (TPT). Abbreviations: DALY, disability-adjusted life year; GDP, gross domestic product.

**Figure 2.**
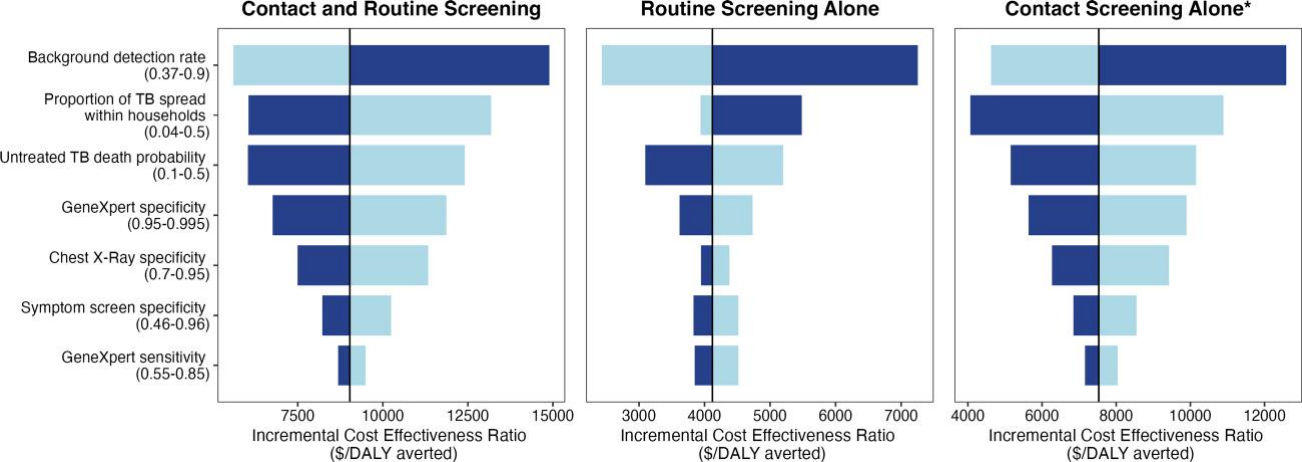
One-way sensitivity analysis of model parameters. Figure displays parameters that change ICER by 10% or more compared with base case in 1-way sensitivity analysis. Light gradation corresponds to parameter values lower than base case, and dark gradation corresponds to parameter values higher than base case. Scenarios include preventive treatment for all household contacts. Abbreviations: ICER, Incremental Cost Effectiveness Ratio; TB, tuberculosis; TPT, tuberculosis preventive treatment. *Contact screening alone is a dominated strategy with the inclusion of other interventions; ICER is compared only to the base case and no other interventions.

The public health impact and cost-effectiveness of age-based routine screening remained robust in probabilistic sensitivity analyses. At a willingness-to-pay threshold of the gross domestic product (GDP) per capita of South Africa ($6414), a strategy including age-based routine screening (either with or without contact screening) and preventive treatment was found to be the optimal strategy for 79% of the simulations. If using a willingness-to-pay threshold of 3× GDP per capita ($19 242), age-based routine screening strategies are optimal for 99.2% of the simulations (Figure 1). In 1-way sensitivity analyses, the background detection rate, the proportion of TB spread within households, and untreated TB death probability had the greatest impact on cost-effectiveness (Figure 2).

**Figure 3.**
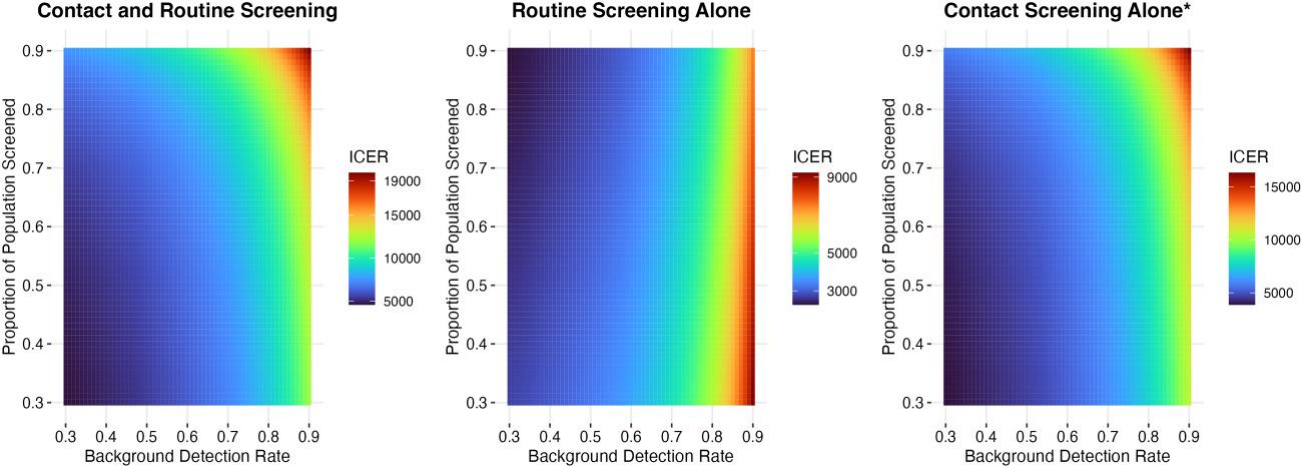
Two-way sensitivity analysis of the proportion of the population screened and background detection rate. Both parameters were varied from 0.3 to 0.9 in increments of 0.01. For all interventions, cost-effectiveness increased (the ICER decreased) for a lower background tuberculosis detection rate. For interventions involving contact screening, cost-effectiveness increased for a lower proportion of the population screened, and for routine surveillance, cost-effectiveness increased for a higher proportion of the population screened. Note that for all scenarios in this sensitivity analysis, the ICER for all interventions reamained cost-effective. Abbreviation: ICER, incremental cost-effectiveness ratio. *Contact screening alone is a dominated strategy with the inclusion of other interventions; ICER is compared only to the base case and no other intervention.

The cost-effectiveness of the interventions also changed with the proportion of the population screened. For interventions involving contact screening, cost-effectiveness increased for a lower proportion of the population screened, and for routine surveillance, cost-effectiveness increased for a higher proportion of the population screened (Figure 3).

## DISCUSSION

In this modeling study, we found that routine screening for TB disease among young children, combined with contact screening, would have a large public health impact in South Africa. It was also found to be cost-effective, even accounting for the considerable uncertainty in estimates of key parameters concerning pediatric TB natural history and epidemiology. Compared with the status quo, all strategies were cost-effective and decreased deaths. Importantly, preventive treatment improved public health impact in all interventions and strategies while remaining cost-effective, indicating the value of this intervention at multiple levels. These results suggest that in a comprehensive approach to the pediatric TB epidemic in high-burden settings such as South Africa, targeting both children exposed to TB cases in the household and all young children could have substantial public health benefits.

Children younger than age 5 years who are exposed to a household TB case have been long recognized as a priority group for TB preventive treatment. In 2020, the World Health Organization expanded its guidance to recommend preventive treatment for TB household contacts of all ages [37]. However, in most high TB-burden countries, preventive treatment coverage for children, particularly older children (aged 5–15 years), who are contacts of TB cases has been modest [2]. Prior modeling studies have found that scaling up contact preventive treatment to child household contacts could have substantial impacts on disease and mortality [4, 6, 38]. Our results are consistent with these studies finding that contact tracing is highly effective at detecting children with TB disease quickly and allowing for implementation of preventive treatment to children without disease [8, 9]. However, given growing evidence that the majority of TB infections in children are acquired outside the household, household-based interventions may be insufficient to fully address the undiagnosed burden of TB in children and to prevent incident cases. Furthermore, a substantial proportion of pediatric TB cases occurring in households with adult cases are already prevalent at the time of contact investigations, which misses the window of opportunity for prevention [11]. To address these limitations, broader screening efforts may be needed in high TB-burden settings, though such screening is associated with substantial costs. We therefore expanded on these modeling analyses by evaluating age-based population-level screening for active disease and infection with targeted preventive treatment. We found that such screening, alongside preventive treatment, could be highly impactful and cost-effective in communities with high TB transmission rates.

As expected, we found that the benefits of case finding and prevention interventions were highly sensitive to the annual risk of TB infection. However, age-based routine screening in combination with contact screening remained cost effective above a force of infection of 1.6%, and age-based routine without increased contact screening remained cost-effective above a force of infection of 0.8%. Most high-burden countries, including South Africa, have an annual risk of TB infection higher than 1.6% [13, 39]. In scenario analysis, the effectiveness of strategies was increased by more than 40% when adding the provision of preventive treatment for individuals with household contacts. This is likely because of the high degree of progression from early TB infection to disease in children [14]. Nearly one-quarter of infected children younger than age 5 years will progress to disease [13, 14]. Therefore, stopping development from TB infection to disease remains a critical component of pediatric TB control.

Our study has several limitations. First, estimates of the annual risk of TB infection in South Africa are heterogenous, in part reflecting the heterogeneity in TB burden across the country. By province, the Eastern Cape had the largest proportion of the entire population who died from TB (8.3%); Gauteng had almost half of that proportion (4.7%) [40]. We used an overall estimate of 4% annual risk of infection [4, 15, 19, 20], and performed broad sensitivity analyses across a range of rates. Furthermore, background TB detection, natural progression of untreated TB, total pediatric TB deaths, and the proportion of TB spread within the household are all highly uncertain parameters [1,4, 11, 13, 19]. Despite this, the cost-effectiveness of age-based routine screening remained robust when we varied values widely in our sensitivity analyses. Second, our model structure uses a constant force of infection, assuming that interventions targeted at children are unlikely to have substantial transmission impacts. This simplification could lead to underestimation of the impact and cost-effectiveness of each intervention. Similarly, we did not include adults or adolescents aged greater than 15 year who would be screened alongside children during contact investigation, a simplification that may underestimate the cost-effectiveness of contact investigations. Third, our model does not consider capital costs associated with scaling up TB interventions at a nationwide level and how overall cost would be managed. These costs may be substantial, and the overall estimated cost of scaling up these interventions was already 17% of the total TB budget for South Africa in 2016–2017 [41]. Fourth, we assumed that all individuals start in the “susceptible” state at birth with homogeneous susceptibility to infection, recognizing that there are heterogeneities in risk of infection and disease progression that could impact the strategies tested. Fifth, a proportion of children may not participate in a population-based TB screening program and the yield may be much lower than our estimated 75% [28]. However, cost-effectiveness remained robust across a range of yields. Sixth, multidrug-resistant TB was not accounted for. It is estimated to account for between 3% and 9% of TB in South Africa, and likely will increase treatment cost and decrease treatment effectiveness [42, 43]. Seventh, we assume that individuals are adherent with screening appointments, such as having tuberculin skin tests read and interpreted. Eighth, our study modeled isoniazid monotherapy for prevention. The field of TB preventive therapy may be moving to isoniazid and rifapentine combination therapy and isoniazid and rifampin therapy for children. These therapies are known to have higher completion rates and less risk of undertreatment but are more costly [44]. Finally, we applied several simplifying model assumptions.

The current global strategy for pediatric TB prevention is centered largely around household contact investigations. Even if scaled up, this strategy may fail to prevent most TB cases in children. Our model suggests that a more proactive strategy of screening all young children, based on age, for active disease and infection, combined with use of preventive treatment, could be cost-effective in high TB-burden settings. Studies will be needed to identify effective, pragmatic approaches to implementation of routine TB screening for young children and to evaluate their impact on prevention. In view of the estimated 200 000 deaths from pediatric TB that continue to occur each year, testing more comprehensive approaches to TB prevention among children should be an urgent priority.

## Supplementary Data

Supplementary materials are available at *Clinical Infectious Diseases* online. Consisting of data provided by the authors to benefit the reader, the posted materials are not copyedited and are the sole responsibility of the authors, so questions or comments should be addressed to the corresponding author.

## Supplementary Material

ciad449_Supplementary_Data

## Notes

***Financial support*.** This work was supported by the Stanford University School of Medicine Medical Scholars Research Program and the National Institutes of Health (NIH R01 143636 to M.H.). N.C.L. is supported by the National Institutes of Health NIAID New Innovator Award (DP2AI170485). L.M. is supported by the National Institutes of Health (K01AI156022 and L40AI140340). The funders of the study had no role in study design, data collection, data analysis, data interpretation, or writing of the report.
