# Supplementary material for: Public Health Impact and Cost-Effectiveness of Screening for Active Tuberculosis Disease or Infection Among Children in South Africa
Source: Clin Infect Dis. 2023 Aug 5;77(11):1544–51. doi: 10.1093/cid/ciad449 (PMC10686943; doi:10.1093/cid/ciad449)
Supplement: ciad449_Supplementary_Data [file ciad449_supplementary_data.docx]

**SUPPLEMENTARY APPENDIX**

**Description of model mechanics**


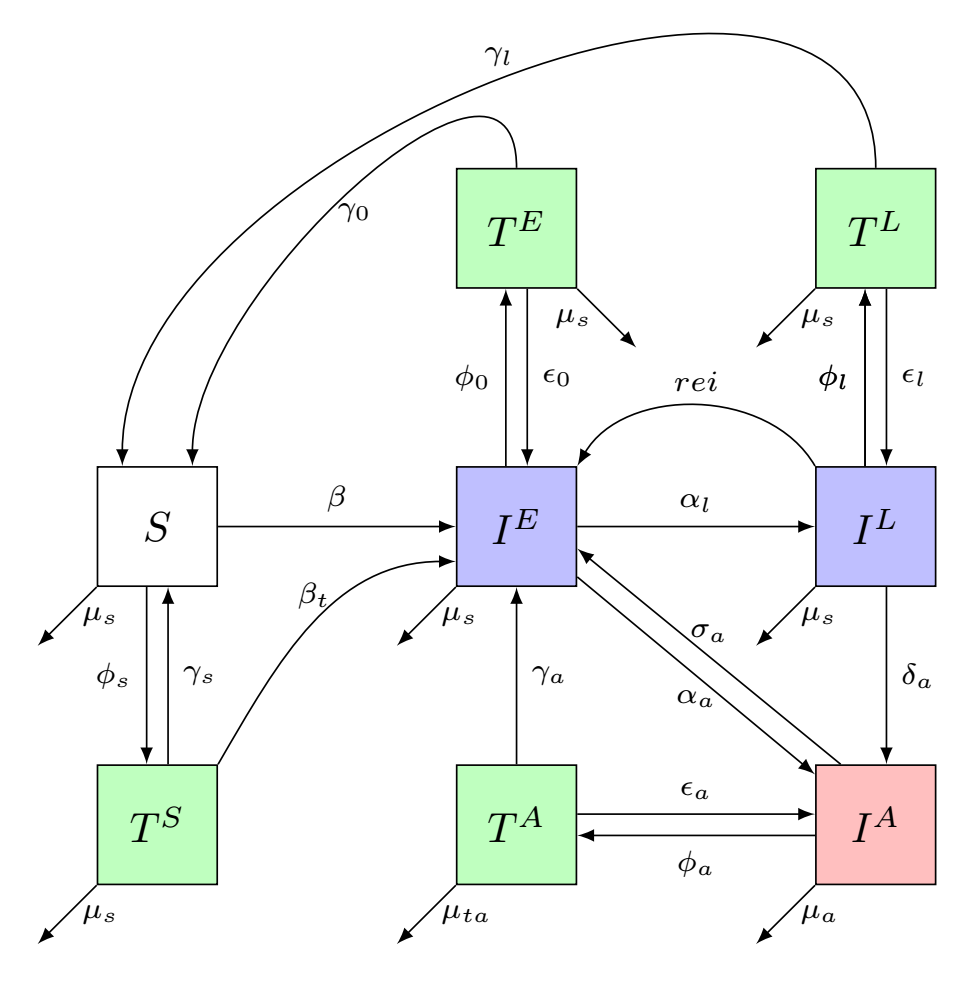


**Figure S1: Illustration of basic mechanics of pediatric TB model.**

This is a deterministic differential equations model. The graphic structure of the model is shown in Figure S1, and the differential equations are displayed in Figure S2. The model consists of four disease states: Susceptible (S), early latent infection (I^E^), late latent infection (I^L^), and TB disease (I^A^). All individuals start in the S state at birth and if infected are moved into I^E^. From I^E^, individuals have a chance of moving to I^A^, and move to I^L^ if they do not progress to TB disease in 12 months of I^E^. Individuals in I^E^ have a much greater chance of progression to I^A^ than individuals in I^L^. TB reinfection can occur by which an individual is infected with a different TB strain while in the I^L^ state, and they will then move back to the I^E^ state (rei).

The only way for an individual to move to the T^S^ state is through a false-positive TB test. The T^S^ state has a lower but nonzero chance of infection. Individuals treated with preventive therapy or TB disease therapy with latent TB move into the T^E^ or T^L^ state, and if successfully treated will move back to the S state. Individuals who recover from TB disease infection with or without treatment move back to the I^E^ state. Children with latent infection who received anti-tuberculosis treatment (e.g., due to false positive testing for disease) were considered to have had their latent tuberculosis treated and placed in the T^L^ or T^E^ state.


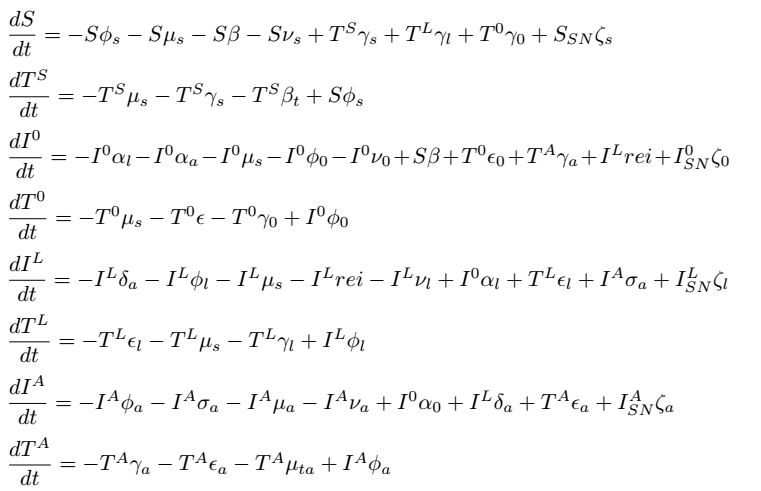


***Figure S2: differential equations used in model***

1.
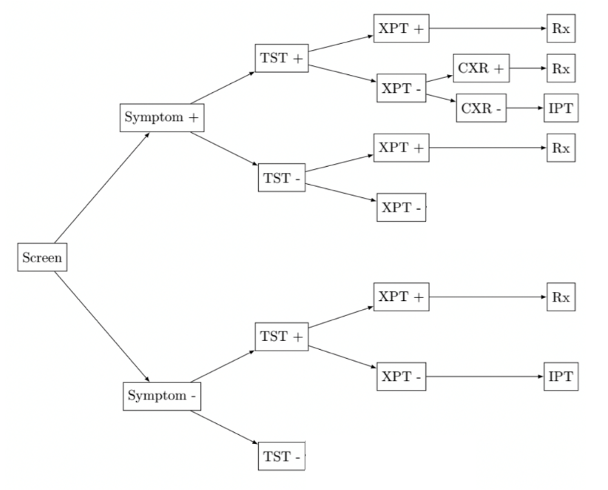

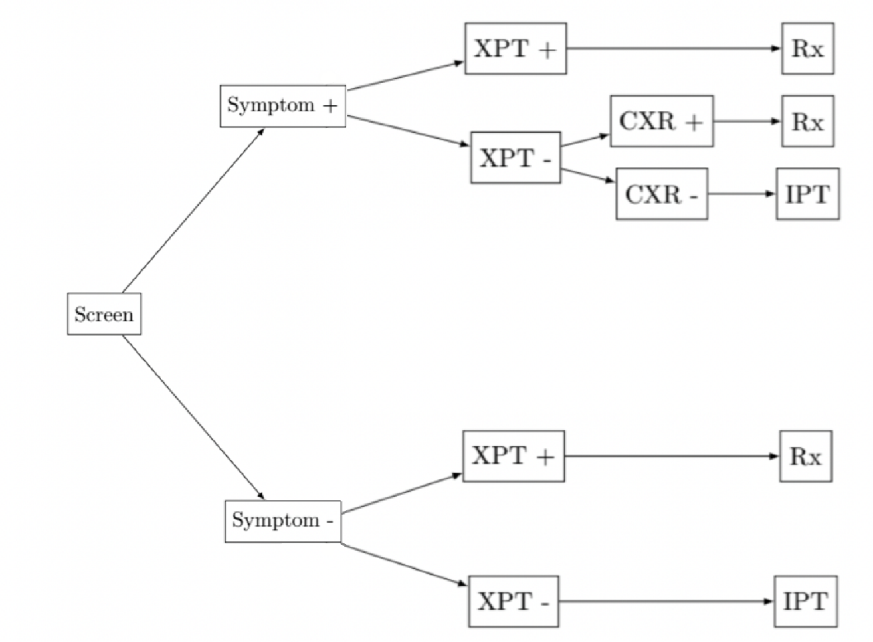
Contact Screening Algorithm B. Age-based Routine Screening Algorithm

**Figure S3: Cascade of care for individuals selected for TB screening.**

A. Individuals undergoing contact screening are first screened for symptoms, then GeneXpert is used. Those symptom positive and Xpert negative undergo chest X-ray. Either Xpert positivity or Chest X-ray positivity confers active TB treatment. All others undergo preventive therapy.

B. Individuals undergoing age-based routine screening are first screened for symptoms, then undergo tuberculin skin test (TST). Those TST positive or symptom positive then undergo GeneXpert. Those TST positive and Xpert positive undergo Chest X-ray. Having either a positive Xpert or Chest X-ray confers active treatment. All other symptom positive individuals and TST positive individuals without symptoms undergo preventive therapy.

Abbreviations: TST = Tuberculin skin test, XPT = GeneXpert, CXR = Chest X-ray, IPT = Isoniazid preventive therapy, Rx = Active TB treatment

**Table S1: Expanded natural history, treatment, diagnostic parameters and costs for tuberculosis screening in children.**

| **Parameter** | **Value** | **Low CI** | **High CI** | **Reference** |
| --- | --- | --- | --- | --- |
| Symptom sensitivity ( <3y HIV-) | 0.518 | 0.44 | 0.74 | [45] |
| Symptom sensitivity (>3 HIV -) | 0.823 | 0.76 | 0.87 |  |
| Symptom sensitivity (HIV +) | 0.562 | 0.41 | 0.71 |  |
| Symptom specificity ( <3y HIV-) | 0.925 | 0.87 | 0.96 |  |
| Symptom specificity (>3 HIV -) | 0.902 | 0.85 | 0.93 |  |
| Symptom specificity (HIV +) | 0.618 | 0.46 | 0.76 |  |
| ATB tx death (0-2) | 0.01 | 0.005 | 0.017 | [4,34] |
| ATB tx death (2-4) | 0.003 | 0.001 | 0.005 |  |
| ATB tx death (5-9) | 0.004 | 0.002 | 0.007 |  |
| ATB tx death (10-14) | 0.0032 | 0.00256 | 0.003 |  |
| ATB tx death (0-2) HIV+ | 0.06861 | 0.02 | 0.179 |  |
| ATB tx death (2-4) HIV + | 0.020605 | 0.007 | 0.057 |  |
| ATB tx death (5-9) HIV + | 0.0274 | 0.010 | 0.072 |  |
| ATB tx death (10-14) HIV + | 0.02192 | 0.011 | 0.039 |  |
| Early latent prog 0-1 | 0.26 | -- | -- | [15] |
| Early latent prog 1-2 | 0.22 | -- | -- |  |
| Early latent prog 2-3 | 0.26 | -- | -- |  |
| Early latent prog 3-4 | 0.2 | -- | -- |  |
| Early latent prog 4-5 | 0.21 | -- | -- |  |
| Early latent prog 5-6 | 0.06 | -- | -- |  |
| Early latent prog 6-7 | 0.12 | -- | -- |  |
| Early latent prog 7-8 | 0.1 | -- | -- |  |
| Early latent prog 8-9 | 0.12 | -- | -- |  |
| Early latent prog 9-10 | 0.11 | -- | -- |  |
| Early latent prog 10-11 | 0.06 | -- | -- |  |
| Early latent prog 11-12 | 0.1 | -- | -- |  |
| Early latent prog 12-13 | 0.12 | -- | -- |  |
| Early latent prog 13-14 | 0.06 | -- | -- |  |
| Early latent prog 14-15 | 0.11 | -- | -- |  |

**Table S2: Public health impact, cost, and incremental cost-effectiveness of tuberculosis case finding and prevention strategies among children in South Africa ages 0-15.**

| **Intervention** | **Total Cost, millions USD** | **Total DALYs, in 1,000s (95% CrIs)** | **Total Deaths**  **(95% CrIs)** | **∆ Cost, millions**  **(95% CrIs)** | **DALYs Averted, 1000s (95% CrIs)** | **ICER** |
| --- | --- | --- | --- | --- | --- | --- |
| Base | 350  (151-505) | 879  (755-1,168) | 31,910  (26,700-44,600) |  |  |  |
| Contact | 409  (212-572) | 871  (750-1,146) | 31,640  (26,500-41,000) | 59  (43-89) | 8  (6-29) |  |
| ARS 0-1 | 479  (290-646) | 856  (748-1,114) | 31,160  (26,400-42,700) | 131  (106-191) | 23  (6-58) |  |
| ARS 1-2 | 481  (294-653) | 851  (746-1,102) | 30,950  (26,400-42,200) | 139  (107-192) | 28  (8-70) |  |
| ARS 2-3 | 488  (304-653) | 845  (744-1,092) | 30,760  (26,300-41,800) | 138  (111-205) | 34  (10-82) | 4,130  (1,940-11,880) |
| ARS 3-4 | 501  (315-665) | 849  (745-1,098) | 30,880  (26,400-42,000) | 151  (121-218) | 30  (9-73) |  |
| ARS 4-5 | 509  (321-673) | 855  (746-1,112) | 31,090  (26,400-42,400) | 159  (127-226) | 24  (8-59) |  |
| CS+ARS 0-1 | 533  (345-710) | 850  (745-1,097) | 30,950  (26,400-42,000) | 183  (154-262) | 29  (9-77) |  |
| CS+ARS 1-2 | 535  (349-709) | 844  (743-1084) | 30,740  (26,300-41,500) | 185  (154-262) | 35  (11-88) |  |
| CS+ARS 2-3 | 541  (358-715) | 839  (741-1073) | 30,550  (26,300-41,000) | 191  (156-278) | 40  (13-100) | 9,050  (2,890-22,920) |
| CS+ARS 3-4 | 553  (367-728) | 844  (742-1080) | 30,670  (26,300-41,200) | 203  (165-291) | 35  (13-92) |  |
| CS+ARS 4-5 | 560  (372-735) | 849  (743-1093) | 30,870  (26,300-41,600) | 210  (170-299) | 30  (12-79) |  |
| *Abbreviations: ARS: Age-based Routine Screening, CS: contact screening, DALY: disability adjusted life year, ICER: incremental cost-effectiveness ratio* | | | | | | |

**Table S3: Public health impact, cost, and incremental cost-effectiveness of tuberculosis case finding and prevention strategies without preventive therapy among children in South Africa ages 0-15.**

| **Strategy** | **Total Cost, Millions USD** | **DALYs averted in 1000s** | **Incremental Cost, Millions USD** | **Incremental Effect (1000s of DALYs)** | **ICER** |
| --- | --- | --- | --- | --- | --- |
| Base | 246 | 0 |  |  |  |
| Contact | 252 | 5 | 6 | 5 | 1,411 |
| ARS 0-1 | 367 | 8 |  |  |  |
| ARS 1-2 | 369 | 12 |  |  |  |
| ARS 2-3 | 382 | 17 |  |  |  |
| ARS 3-4 | 388 | 18 |  |  |  |
| ARS 4-5 | 392 | 15 |  |  |  |
| C+ARS 0-1 | 373 | 12 |  |  |  |
| C+ARS 1-2 | 376 | 16 |  |  |  |
| C+ARS 2-3 | 387 | 21 | 141 | 16 | 8,390 |
| C+ARS 3-4 | 394 | 21 | 7 | 0.7 | 8,400 |
| C+ARS 4-5 | 397 | 19 |  |  |  |
| *Abbreviations: ARS: Age-based Routine Screening, CS: contact screening, DALY: disability adjusted life year, ICER: incremental cost-effectiveness ratio* | | | | | |
